# Efficacy of manual hyperinflation on arterial blood gases in patients with ventilator-associated pneumonia

**DOI:** 10.1186/s43161-020-00006-8

**Published:** 2020-08-12

**Authors:** Basant H. Elrefaey, Mohamed S. Zidan

**Affiliations:** 1 Department of Physical therapy for Cardiovascular/Respiratory Disorder and Geriatrics, Faculty of Physical Therapy, Cairo University, 7 Ahmed Alzayate Street, Been Alsarayat, Giza, 12111 Egypt; 2 Department of Medical Rehabilitation, Faculty of Applied Medical Sciences, King Khalid University, Abha, Kingdom of Saudi Arabia; 3 Faculty of Physical Therapy, Modern Technology and Information University, Cairo, Egypt

**Keywords:** Arterial blood gases, Manual hyperinflation, Position, Ventilator-associated pneumonia

## Abstract

**Background:**

Tracheal intubation exposes mechanically ventilated patients to serious pulmonary complications such as ventilator-associated pneumonia (VAP). This study was conducted to compare the efficacy of manual hyperinflation in supine versus lateral decubitus position on arterial blood gases (ABG) in patients with VAP. Forty-two patients with ventilator-associated pneumonia with age range from 40 to 60 years were selected. They were randomly divided into two equal groups: group A who received manual hyperinflation from supine position and group B who received manual hyperinflation from lateral decubitus position (upper most affected). Both groups received respiratory physiotherapy. The patients received 2 sessions per day for 6 days. Outcome measures were arterial blood gases (PaO_2_, PaCO_2_, PaO_2_/FiO_2_, and SaO_2_). They were assessed before the 1st morning session (pre), at day 3 (post 1), and at day 6 (post 2).

**Results:**

After sessions, significant changes of measured variables (PaO_2_, PaCO_2_, PaO_2_/FiO_2_, and SaO_2_) were obtained in both groups (*P* < 0.05, in all variables) and by comparison between groups post-intervention; a significant difference was observed between both groups in measures of oxygenation in favor of group B (*P* < 0.05), whereas there was a non-significant difference in the PaC02 between both groups (post 1 *P* = 0.52 and post 2 *P* = 0.33).

**Conclusion:**

It was concluded that effect of the bag squeezing on arterial blood gases in patients with ventilator-associated pneumonia from lateral decubitus position was more effective than from supine position.

**Trial registration:**

PACTR, PACTR201909817075549. Registered October 21, 2018—retrospectively registered

https://pactr.samrc.ac.za/TrialDisplay.aspx?TrialID=4655

## Background

Critically ill adults are often need a machine to help maintain their breathing. An increased risk of ventilator-associated pneumonia (VAP) is one side effect of these machines [1]. Ventilator-associated pneumonia (VAP) can be defined as hospital-acquired pneumonia that develops in intubated patients who have been receiving mechanical ventilation for at least 48 h [2]. In critically ill patients, VAP is associated with increased mortality, increased length of stay in the intensive care unit (ICU), and increased healthcare burden [1].

Physiotherapy is an essential part of the multidisciplinary team in the critical care unit. In mechanical ventilated patients, physiotherapy facilitates removal of excessive airway secretions which in turn decreases airway resistance. It also enhances lung compliance and reduces the work of breathing [3]. Several respiratory physiotherapy techniques, such as body positioning, chest mobilization, percussion, vibrations, and bag squeezing or manual hyperinflation (MHI) are used in mechanically ventilated patients [4].

One of the commonly used respiratory physiotherapy techniques in mechanically ventilated patients is MHI [5]. It has a positive effect on clearance of airway secretion and alveolar recruitment [6]. The delivery of MHI includes a larger than baseline tidal volume (up to one and one half the size of tidal volumes delivered by the ventilator) at a low inspiratory flow (attained by a slow compression of the manual ventilation bag), an inspiratory pause (to allow complete distribution of the inflated air among all the ventilated parts of the lung), and an expiration with a high expiratory flow [7, 8].

Positioning means the usage of body position as a treatment procedure [9]. Positioning for ICU patients has many physiological goals. It enhances oxygen transport through its effects of improving ventilation/perfusion ratio, increasing lung volumes and capacities, decreasing the breathing work, enhancing mucus clearance, and prevents pressure ulcer [10].

In the supine position, closing capacity may encroach on the functional residual capacity resulting in airway closure in dependent lung regions and atelectasis. Airway resistance rises, mostly resulting from abdominal and chest wall compression and in addition, there is an increase in pulmonary blood volume. As a result, lung compliance reduces, ventilation/perfusion mismatches, and the breathing work increases [6].

The side to side positioning has many benefits. It increases the lung volume to the uppermost lung that helps airways clearance of secretions, improves recruitment, and may enhance gas exchange [5]. It also decreases the incidence of VAP provided that more than 40° of lateral turn is achieved [11].

Although manual hyperinflation plays an important role in the multimodal approach to patients in most ICUs, the interpretation and synthesis of results of studies examining the effects of MHI have been limited by differences in definition, dosage, and technique. Only scarce data are available as regards the effect of manual hyperinflation from different positioning on arterial blood gases; also, there is controversy in literature regarding its effect on oxygenation and therefore the present study aimed to compare manual hyperinflation from supine versus side position on arterial blood gases in patients with ventilator-associated pneumonia.

## Methods

### Trial design

This study is a double blind, parallel group, randomized clinical trial. Forty-two mechanically ventilated patients who suffered from ventilator-associated pneumonia were randomly assigned into two groups: the group A and the group B. The group A received manual hyperinflation from supine lying in addition to respiratory physiotherapy, and the group B received manual hyperinflation from side lying (upper most affected) in addition to respiratory physiotherapy. Patients in both groups received manual hyperinflation in addition to respiratory physiotherapy for 20 min, two times per day for a period of 6 days. The study was conducted from August 2017 to March 2018.

### Participants

This study was conducted at Intensive Care Unit, Kasr El-Aini Hospital, Faculty of Medicine, Cairo University, where the patients were recruited. Forty-two mechanically ventilated patients with unilateral lung disease due to ventilator-associated pneumonia participated in this study. They were intubated using endotracheal intubation and received mechanical ventilation in the pressure control ventilation mode with an inspired oxygen fraction of 40%. All patients were on positive end-expiratory pressure (PEEP) 3–5 cmH_2_O. All patients were not operable. The inclusion criteria were mechanically ventilated patients for more than 3 days with ventilator-associated pneumonia according to chest x-ray, ages ranged from 40 to 60 years, and hemodynamic stability. The exclusion criteria were contraindications to assume different positions, bilateral lung dysfunction, repeated mechanical ventilation, unstable cardiovascular conditions, e.g., arrhythmia, and intercostal catheter with a visible air leak. The patients were diagnosed and referred by a physician.

After completion of the initial assessments, the patients who matched the inclusion criteria were randomized to the group A (*n* = 21) or the group B (*n* = 21) using computer-generated block randomization. Allocation concealment was done by opaque envelopes.

The aim and nature of the study were explained for a family member of each candidate before starting the study.

An informed written consent was obtained from a patient’s family member before enrollment. This study was approved by the Institutional Review Board of the Faculty of Physical Therapy, Cairo University (P.T.REC/012/001604). The current study adheres to CONSORT guidelines.

### Evaluative procedures

Study candidates were initially evaluated by the following:

### Chest x-ray

Chest x-ray was used to evaluate lung fields and it was done routinely for all patients who admitted to acute care facilities.

### Auscultation

Stethoscope (2203, Classic II S.E, Littmann stethoscope, USA) was used for auscultation.

### Arterial blood gases analysis

Arterial blood gases analyzer (Abbott Laboratories Pharmaceutical Company, Singapore) was used to measure arterial blood gases before the 1st morning session at day 1 (pre). Arterial blood gas measurement was repeated at day 3 (post 1) and day 6 (post 2) of the study. Blood gases assessment included are as follows: partial pressure of oxygen (PaO_2_), partial pressure of carbon dioxide (PaCO_2_), oxygen saturation (SaO_2_), and ratio of partial pressure of oxygen and fraction of inspired oxygen (PaO_2_/FiO_2_). All outcome measures were taken by an independent assessor who was blinded to group allocation.

### Intervention

All patients continued any current prescribed medications and physiotherapy throughout the trial. The patients were classified into two groups. The patients of group A received manual hyperinflation in the supine position, while the patients of the group B received manual hyperinflation in side-lying position with upper most affected according to chest x-ray.

### Manual hyperinflation

Manual resuscitation bag (size: adult and REF no. 1610-0003, Fortune Medical Instrument CORP, Taiwan) was used for manual hyperinflation. During the entire procedure, all precautions were undertaken to prevent infection to the patient. Heart rate, electrocardiogram, arterial blood pressure (invasive), arterial blood oxygen saturation, respiratory rate, and temperature were monitored using bedside monitor (CARESCAPE Monitor B650, Finland).

1. The patient and bedside monitor were carefully observed to ensure absence of signs of distress, and the position of the endotracheal tube.
2. FiO_2_ was increased to reach 100% for 2 min on mechanical ventilation before disconnecting the patient from mechanical ventilation.
3. The resuscitation circuit was attached to oxygen flow meter (100% O_2_) at a flow rate of 15 L/min, and the bag valve resuscitation circuit was locked at pressure = 40 cmH_2_O.
4. The patients were disconnected from the ventilator; the resuscitation circuit was attached to the filter and to the patient airway (endotracheal).
5. The manual hyperinflation breath was given as the following: a slow inspiratory flow for 3-s duration, followed by a 3-s inspiratory hold, and followed by a fast uninterrupted expiration that simulates a forced expiration [6].
6. The rate of inflation was 10 breaths per minute. The patients had received manual hyperinflation using bag valve resuscitation circuit by the 2-handed technique.
7. Termination of manual hyperinflation occurred, if the patient became cardiovascular unstable or if oxygen saturation dropped.

### Respiratory physiotherapy

Respiratory physiotherapy in the form of percussion, vibration, positioning, and suction were applied for all patients in both groups.

### Data analysis

In this study, the descriptive statistics and *t* test were conducted for comparison of the demographic characteristics between both groups. Mixed MANOVA was conducted to compare the effect of time (pre versus post) and the effect of treatment (between groups), as well as the interaction between time and treatment on mean values of arterial blood gases. Post hoc tests using the Bonferroni correction were carried out for subsequent multiple comparisons. Chi-squared test were conducted for comparison of diagnosis distribution between both groups. The level of significance for all statistical tests was set at *P* < 0.05. All statistical measures were performed through the statistical package for social studies (SPSS) version 19 for windows (IBM Corp., Armonk, NY, USA).

The demographic data and clinical outcomes were expressed as the mean (SD) [i.e., age, weight, height, body mass index (BMI), PaO_2_, PaCO_2_, SaO_2,_ and PaO_2_/FiO_2_]. The frequency distribution and chi-squared test were used for comparison of distribution of diagnosis in both groups.

### Sample size

Sample size calculation was performed prior to the actual study based on results of pilot study using the G*POWER statistical software (version 3.1.9.2; Franz Faul, Universitat Kiel, Germany) and revealed that the appropriate required sample size was 40. The primary outcome measure was PaO_2_. The calculations were made using *α* = 0.05, *β* = 0.2, and large effect size.

## Results

**Fig. 1:**
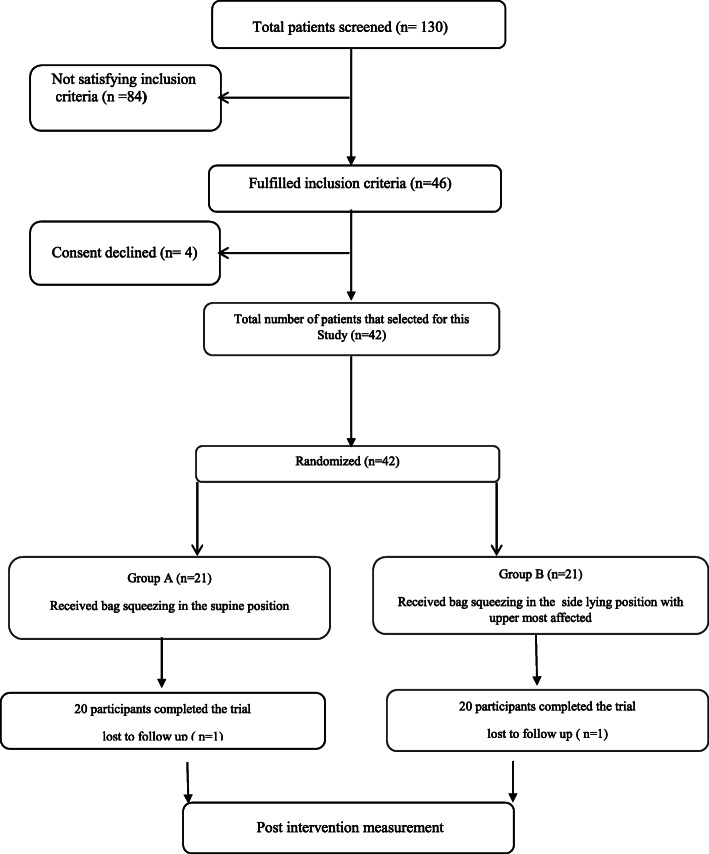
Flow diagram of the study

Both groups were homogenous in their demographic data and in the baseline clinical outcome measures (Table 1). Overall, 130 patients were screened. Of which, 84 not fulfilled the inclusion criteria and 4 patients declined to participate, so 42 patients were randomized. Forty patients (20 in the study group and 20 in the control group) completed the duration of the treatment as unfortunately two patients died (one in each group) as shown in Fig. 1. The analysis was accomplished on participants who finished all sessions, along with the pre- and post-intervention assessments. There was a significant interaction effect of group and time (*P* = 0.0001). There was a significant main effect of group (*P* = 0.0001) and a significant main effect of time (*P* = 0.0001).

**Table 1:** Demographic and baseline clinical characteristics of patients in both groups

Characteristics	Group A (supine-lying position) (*n* = 20)	Group B (side-lying position) (*n* = 20)	*P* value
Age (years)	48.2 ± 6.38	51.85 ± 7.31	0.1
Weight (kg)	82.2 ± 6.17	80.25 ± 6.67	0.34
Height (cm)	173.45 ± 4.72	172.8 ± 5.13	0.67
BMI (kg/m^2^)	27.32 ± 1.71	26.88 ± 2.17	0.48
Side of ventilator-associated pneumonia			
Right	14 (17%)	16 (87%)	0.46
Left	6 (30%)	4 (13%)	
PaO_2_ (mmHg)	79.3 ± 5.01	81.16 ± 4.96	0.24
PaCO_2_ (mmHg)	40.8 ± 3.66	42.37 ± 2.96	0.14
SaO_2_ (%)	94.15 ± 1.03	94.5 ± 0.9	0.26
PaO_2_/FiO_2_	206.93 ± 8.46	206.44 ± 11.33	0.87
PH	7.38 ± 0.03	7.37 ± 0.02	0.13

Values are presented as mean ± standard deviation or number (%) (in case of side of ventilator-associated pneumonia)

*BMI* body mass index, *PaCO*_*2*_ partial pressure of carbon dioxide, *PaO*_*2*_ partial pressure of oxygen, *PaO*_*2*_*/FiO*_*2*_ ratio of partial pressure of oxygen and fraction of inspired oxygen, *SaO*_*2*_ oxygen saturation

Regarding within-group comparisons, both groups revealed significant results in all outcomes (*P* < 0.05) (Table 2).

**Table 2:** Comparison of arterial blood gases between group A and B and between pretreatment, post 1, and post 2 in each group

Parameters	Group A (*n* = 20) (supine-lying position)				Group B (*n* = 20) (side-lying position)				Between-groups *P* values		
	Pre	Post 1	Post 2	*P* value	Pre	Post 1	Post 2	*P* value	Pre	Post 1	Post 2
PaO_2_ (mmHg)	79.3 ± 5.01	93.16 ± 10.26	119.06 ± 8.84	0.0001^*^	81.16 ± 4.96	114.9 ± 18.77	142.15 ± 13.46	0.0001^*^	0.24	0.0001^*^	0.0001^*^
PaCO_2_ (mmHg)	40.8 ± 3.66	38.66 ± 3.62	36.58 ± 3.18	0.0001^*^	42.37 ± 2.96	39.32 ± 2.91	35.71 ± 2.38	0.0001^*^	0.14	0.52	0.33
SaO_2_ (%)	94.15 ± 1.03	95.45 ± 0.99	96.96 ± 1.19	0.0001^*^	94.5 ± 0.9	97.17 ± 0.83	99.07 ± 0.66	0.0001^*^	0.26	0.0001^*^	0.0001^*^
PaO_2_/FiO_2_	206.93 ± 8.46	247.58 ± 25.32	330.27 ± 27.56	0.0001^*^	206.44 ± 11.33	300.16 ± 41.14	386.81 ± 21.17	0.0001^*^	0.87	0.0001^*^	0.0001^*^

Values are presented as mean ± standard deviation

*PaCO*_*2*_ partial pressure of carbon dioxide, *PaO*_*2*_ partial pressure of oxygen, *PaO*_*2*_*/FiO*_*2*_ ratio of partial pressure of oxygen and fraction of inspired oxygen, *SaO*_*2*_ oxygen saturation

*Significant

Pre versus post 1 intragroup comparisons for PaO_2_, SaO_2_, and PaO_2_/FiO_2_ showed in group A significant increases, by 13.86 mmHg, 1.3%, and 40.65, while for PaCO_2_, it showed significant decrease by 2.12 mmHg. Group B also showed significant increases, by 33.7 mmHg, 2.67%, and 93.7, respectively, while for PaCO_2_, it revealed significant decrease by 3.05 mmHg. For the intergroup comparisons, PaO_2_, SaO_2_, and PaO_2_/FiO_2_ showed significant differences between both groups (*P* = 0.0001 in all comparisons) favoring group B (side-lying position), but PaCO_2_ showed non-significant difference at post 1 measurement (*P* = 0.52).

Pre versus post 2 within-group comparisons for PaO_2_, SaO_2_, and PaO_2_/FiO_2_ showed in group A significant increases, by 39.76 mmHg, 2.81%, and 123.34, while for PaCO_2_, it showed significant decrease by 4.2 mmHg. Group B also showed significant increases, by 61 mmHg, 4.57%, and 180.37, respectively, while for PaCO_2_, it showed significant decrease by 6.66 mmHg. For the intergroup comparisons, PaO_2_, SaO_2_, and PaO_2_/FiO_2_ showed significant differences between both groups (*P* = 0.0001 in all comparisons) favoring group B (side-lying position), but PaCO_2_ showed non-significant difference at post 2 measurements (*P* = 0.33) (Table 2).

## Discussion

Patients in intensive care unit are vulnerable to complications due to different reasons (underlying disease, immobilization, infection risk…). The current main intervention in order to prevent these complications is respiratory physiotherapy. This study compared the effects of manual hyperinflation on arterial blood gas values, which included PaO_2_, PaCO_2_, SaO_2_, and PaO_2_/FiO_2_ in ventilator-associated pneumonia patients from 2 different positions. To the best of our knowledge, this study is the first study to investigate the effects of manual hyperinflation from supine versus side-lying position on arterial blood gases of ventilator-associated pneumonia patients.

The results of the present study revealed that there was a significant change of PaO_2_, SaO_2_, PaCO_2_, and PaO_2_/FiO_2_ in both groups at post 1 and post 2 compared with pretreatment. However, manual hyperinflation from side lying was superior to manual hyperinflation from supine regarding PaO_2_, SaO_2_, and PaO_2_/FiO_2_ with non-significant difference in PaCO_2_ between groups.

The results of the current study are supported by previous trials of Berney et al. [12], Savian et al. [13], and Dennis et al. [14]. They supported the application of manual hyperinflation with the patient in a side-lying position with the affected side uppermost in artificially ventilated and intubated intensive care patients. The side-lying position has many advantages. It increases the lung volume of the upper lung which improves recruitment and helps bronchopulmonary segment drainage and it may also enhance gas exchange and ventilation-perfusion ratio [5]. All of this may explain the positive changes observed in the current study. The addition of a side-lying position was superior to the supine position in sputum production when using MHI which also in line with the current study [14].

The improvement in oxygenation is supported by Blattner et al. [15] and Paulus et al. [16]. They recommended manual hyperinflation for intubated and mechanically ventilated critically ill as it may improve pulmonary compliance, arterial oxygenation, and clearance of airway secretions. Ahmed et al. [17] found that the PaO_2_/FiO_2_ ratio improved significantly at both 1 and 20 min posttreatment for MHI in patients undergoing mitral valve replacement. In a similar study, Patman et al. [18] investigated effects of MHI on PaO_2_/FiO_2_ in patients within 4 h of coronary artery bypass graft surgery. They examined the effect of MHI alone versus no MHI, and the result showed greater improvement in PaO_2_/FiO_2_ ratio in all cases of MHI group, similar to the results of our study. However, they showed that there was not change of SaO_2_ between the two groups.

In contrast, Hodgson et al. [5] did not find any differences between the addition of manual lung hyperinflation to side-lying treatment and side-lying treatment alone for gas exchange (PaO_2_/FIO_2_ and PaCO_2_) in critically ill patients. Also, Paulus et al. [19] and Paulus et al. [20] concluded that MH did not affect oxygen saturation. Dennis et al. [21] found that it deteriorated over time with MHI. This controversy may be due to different diagnoses (VAP) and different time of the session and different techniques.

Moreover, Barker and Adams [22] investigated mechanically ventilated patients with acute lung injury. Patients were randomized into one of three treatment groups: group 1 (suctioned only), group 2 (positioned and suctioned), and group 3 (positioned, manually hyperinflated, and suctioned). Their results demonstrated significant changes in PaCO_2_ over time for all three groups which support current study results. However, (PaO_2_/FiO_2_) did not alter significantly in any of the groups.

The current study was supported by Hariedy et al. [23] who examined the effect of chest physiotherapy (MHI, suction, percussion, and vibration) on arterial blood gases in mechanically ventilated patients with acute lung injury. The result showed significant changes in PaO_2_, SaO_2_, and PaCO_2_ in the study group compared to the control group.

Also, Soundararaian and Thankappan [24] and Malekzadeh et al. [25] examined the effect of manual hyperinflation on PaO_2_, SaO_2_, and PaCO_2_ in different populations. Their results showed that there were significant changes which also support current results.

This present study had some limitations. The ABG effects were measured only post the intervention. Jones et al. [26] had been described that physiotherapy-induced improvements can last for up to 2 h after intervention. Also, this study was a single-center study, so multicenter study is recommended with a larger sample size. The findings of the present study may not be generalized to all various available manual resuscitation bags because variability exists in the effects of different bags. This study was a short-term study, so time to resolution of the pneumonia, time of extubation, time of mechanical ventilation, and ICU length study were not measured.

Further research is needed to determine the long-term effects of manual hyperinflation on mechanically ventilated patients with VAP from different decubitus and its effect on weaning from mechanical ventilation and resolution of infection. The prophylactic use of this technique from different decubitus in preventing VAP is also an area of future research.

## Conclusion

In conclusion, it would appear from this study that manual hyperinflation has a great effect on arterial blood gases on both groups (supine group) and (side group), but its effect was greater with positioning of patients according to their pathology in side-lying position,

## Data Availability

The dataset generated during the current study are available from the corresponding author on a reasonable request.

## Abbreviations

ABG: Arterial blood gases
BMI: Body mass index
ICU: Intensive care unit
MHI: Manual hyperinflation
PaO_2_: Partial pressure of oxygen
PaCO_2_: Partial pressure of carbon dioxide
PaO_2_/FiO_2_: Ratio of partial pressure of oxygen and fraction of inspired oxygen
PEEP: Positive end expiratory pressure
SaO_2_: Oxygen saturation
VAP: Ventilator-associated pneumonia
